# Results of thermal osteonecrosis for implant removal on electron microscopy, implant stability, and radiographic parameters – a rat study

**DOI:** 10.1186/s13005-023-00349-2

**Published:** 2023-03-07

**Authors:** Kristian Kniha, Eva Miriam Buhl, Faruk Al-Sibai, Stephan Christian Möhlhenrich, Anna Bock, Marius Heitzer, Frank Hölzle, Ali Modabber

**Affiliations:** 1 Department of Oral and Cranio-Maxillofacial Surgery, University Hospital RWTH, Pauwelstraße 30, Aachen, Germany; 2 Private Clinic for Oral Surgery Dres. Kniha, Rosental 6, 80331 Munich, Germany; 3 Institute for Pathology, Electron Microscopy Facility, University Hospital RWTH, Pauwelstraße 30, 52074 Aachen, Germany; 4 Institute of Heat and Mass Transfer, University Hospital RWTH, Augustinerbach 6, Aachen, Germany; 5 Department of Orthodontics, University of Witten/Herdecke, Alfred-Herrhausen Str. 45, 58455 Witten, Germany

**Keywords:** Osteocyte lacuna, Osteonecrosis, Temperature, Histopathology

## Abstract

**Background:**

This rat study aimed to evaluate the feasibility of temperature thresholds that affect peri-implant bone cells and morphology and the potential usefulness of thermal necrosis for inducing implant removal for a subsequent in vivo pig study.

**Methods:**

On one side, rat tibiae were thermally treated before implant insertion. The contralateral side was used as the control group without tempering. Temperatures of 4 °C, 3 °C, 2 °C, 48 °C, 49 °C, and 50 °C were evaluated with a tempering time of 1 min. Energy-dispersive X-ray spectroscopy (EDX) and transmission electron microscopy (TEM) analyses were performed.

**Results:**

The EDX analysis revealed significant increases in element weights at 50 °C (e.g., calcium, phosphate, sodium, and sulfur; *p* < 0.01). The results of the TEM analysis showed that at all the applied cold and warm temperatures, signs of cell damage were observed, including vacuolization, shrinkage, and detachment from the surrounding bone matrix. Some cells became necrotic, leaving the lacunae empty.

**Conclusions:**

Temperature of 50 °C led to irreversible cell death. The degree of damage was more significant at 50 °C and 2 °C than at 48 °C and 5 °C. Although this was a preliminary study, from the results, we identified that a temperature of 50 °C at a time interval of 60 min can lower the number of samples in a further study of thermo-explantation. Thus, the subsequent planned in vivo study in pigs, which will consider osseointegrated implants, is feasible.

**Supplementary Information:**

The online version contains supplementary material available at 10.1186/s13005-023-00349-2.

## Introduction

In the current literature, only few data support the use of a controlled thermoexplantation procedure for implant removal. No clear indications have been established on the exact temperature and time interval that would produce sufficient but minimal osteonecrosis around implants to remove them without trauma. Uncontrolled temperatures could easily lead to extensive jaw necrosis and would be more likely to cause severe inflammation rather than atraumatic explantation. Consequently, a precise temperature within the initial developmental range of bone necrosis must be selected for successful thermal explantation. Nevertheless, several publications have demonstrated the efficient loosening of osseointegrated implants using warm temperatures with, for example, ultra-high-frequency and laser surgical devices [1–3]. The heat input was uncontrolled and inconsistent in all the reported cases.

A recent systemic review analyzed the literature regarding the threshold values for thermal bone necrosis [4]. The authors concluded that no studies have indicated a specific threshold value for bone necrosis. For a tempering time of 1 min, the values ranged from 47 °C to 55 °C, but no threshold value has yet been established for cryoinsult. Thus, in the current literature, no clear evidence exists that support the use of a managed thermo-explantation technique or suggest the exact temperature and time interval around the implants that would generate adequate but minimal osteonecrosis to extract them without trauma. Dental implants that replace failed implants have lower survival rates. However, if they osseointegrate successfully, the method described here can be considered [5]. High temperatures could easily lead to substantial jaw necrosis and cause severe inflammation. Therefore, more in-depth and pre-clinical studies are needed to gain further insights to evaluate the potential usefulness of thermal necrosis for implant removal before it can be predictably applied to patients. In our preliminary cadaver study, temperatures of 51 °C and 5 °C at time intervals of 10 and 30 s were identified, respectively, which led to significant osseous matrix degeneration [6]. In addition, transmission electron microscopy (TEM) at 53 °C revealed a decalcification and swollen mitochondria, which lost the structure of their inner cristae.

The present rat study was conducted to verify these results in an in vivo setting and reduce the sample size for subsequent in vivo studies in the pig with osseointegrated implants. The primary aim of this study was to analyze feasible cold and warm temperatures and time intervals. For this purpose, mineralization of the peri-implant bone of the rat tibia was analyzed on energy-dispersive X-ray spectroscopy (EDX), and osteocyte morphology was analyzed for signs of injury on TEM. We hypothesized that an appropriate temperature and time interval for warm and cold temperatures would induce minimal peri-implant osteonecrosis. Furthermore, implant stability and bone loss over time were measured.

## Methods

### Experimental protocol

This study included 48 adult male Sprague–Dawley rats, each weighing 450 g and aged 4 months (Janvier Labs, Le Genest-Saint-Isle, France). One examiner completed all the steps of the study experiment. This investigation was conducted in accordance with the guidelines of the European Parliament and the council on the protection of animals used for scientific purposes, ARRIVE (Animal Research: Reporting of In Vivo Experiments) [7], and Directive 2010/63/EU. The study protocol received ethical approval and consent to participate from the appropriate local authority (Landesamt für Natur und Verbraucherschutz, Recklinghausen, Germany; Ref. 2019A276).

For a tempering time of 1 min, the six test groups were divided into cold and warm temperatures of 4 °C, 3 °C, 2 °C, 48 °C, 49 °C, and 50 °C. These temperatures were chosen based on the findings of a prior investigation [8]. In each group, six temperatures were randomly tested in eight animals. Each animal received one implant in each tibia (Modus 2.0 3-mm cortical screws, Medartis AG, Basel, Switzerland). Therefore, for each temperature, eight test und eight control samples were investigated, resulting in 12 groups with 96 implants. All the procedures were performed under general anesthesia. At 30 min before the start of surgery, the animals were weighed. Then, the animals received 0.03-mg buprenorphine subcutaneously (0.1-ml Temgesic/kg of body weight, Remedix GmbH, Germany). Anesthesia was induced with an inhalation narcotic in an induction box with isoflurane (4 vol.%) and oxygen (vol. 30%) air mixture (Isofluran, Piramal GmbH, Hallbergmoos, Germany). Inhalation narcosis with an isoflurane (2 vol.%) and oxygen (30%) air mixture was continued via a nasal mask. After the induction of anesthesia, the animals were positioned on an adjustable warming mat.

**Fig. 1:**
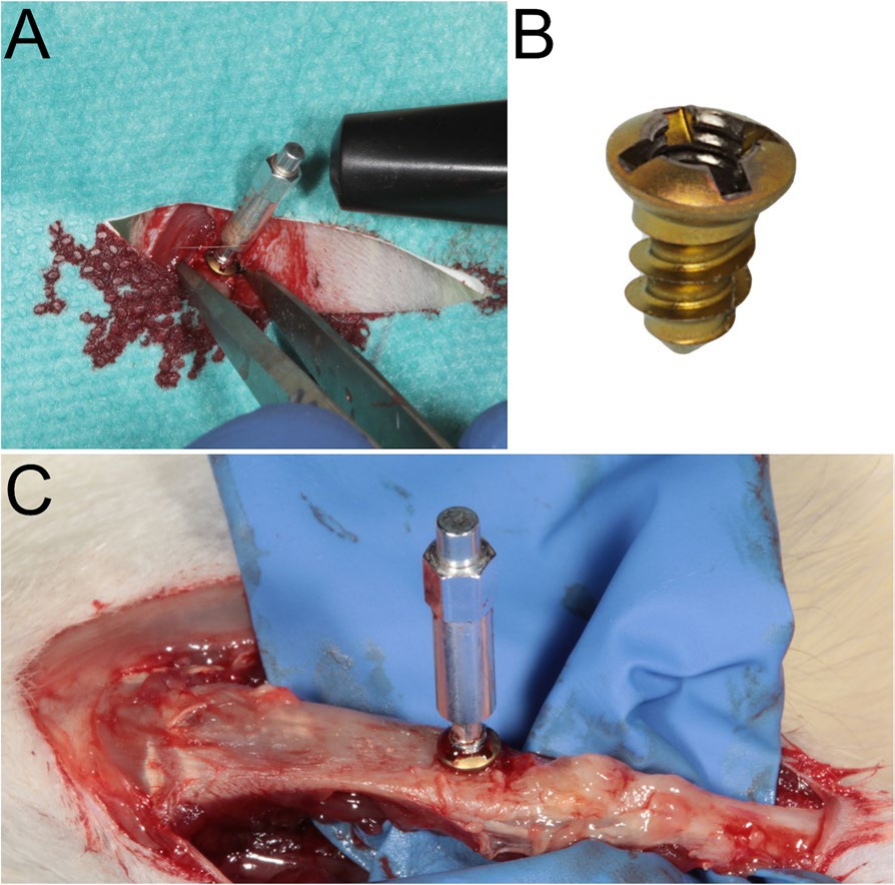
**A** After implant insertion, the ISQ values were measured for the first time. **B** The mini-screws used. For the ISQ values, threads were cut on the head of the screw. **C** The second implant stability measurement was performed after 7 days

**Fig. 2:**
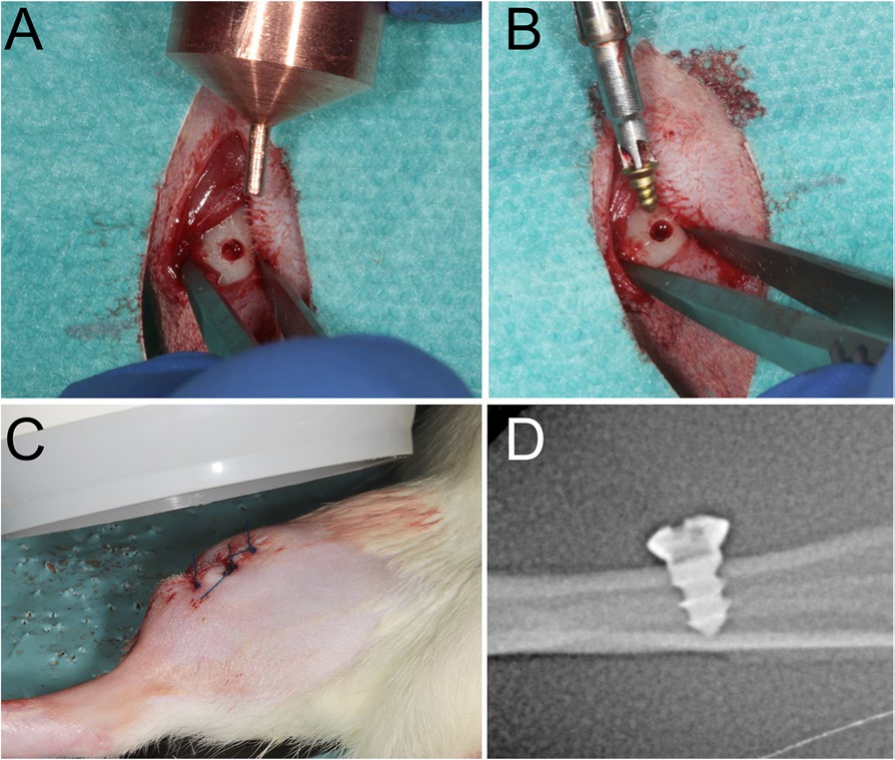
**A** In the case of the test group, the thermal treatment was administered after predrilling. The surface of the device individually tempered the bone surface **B** After the thermo-treatment, implantation was performed. **C** The radiographic evaluation was performed directly after the surgery and after 7 days. **D** A standardized radiographic measurement procedure was used to measure the crestal bone changes around the implant body

Before implant insertion, one tibia was randomly chosen as the test side in each animal. Without tempering, the contralateral side was used as the control group. Before temperature application and implantation, the skin was shaved, disinfected, and cut with a scalpel after sterile draping. Implantation was performed after exposure of the tibia by predrilling with a pilot drill of 1.5-mm diameter under strict cooling with sterile saline (Medartis AG, Basel, Switzerland). In the case of the test group, the thermal treatment was administered after predrilling and before implant placement. The device exhibited a congruent clamp fit in the drill studs. A screw was inserted in accordance with the manufacturer's protocol by using a screwdriver and a torque of 15–20 Ncm. After primary stable insertion of the implants, resonance frequency analysis with hand-screwed individual smart pegs (Osstell, Gothenburg, Sweden) was performed to measure the primary stability after implant insertion (Fig. 1). Primary stability was measured with the implant stability quotient (ISQ) in four directions (i.e., from left to right and from front to back [9]), resulting in a calculated mean ISQ value. The wounds were closed with suture material. By using a standardized 90° dental X-ray computed tomography device (Dentsply Detrey, Germany, Konstanz), the distance between the alveolar crest at the implant and the implant shoulder was measured directly after surgery on both implant sides [10]. The measurements were taken between the implant shoulder and the greatest coronal level of the direct bone-to-implant contact (Fig. 2). All the measurements were performed using specialized computer software (ImageJ Version 1.51, National Institutes of Health, USA) [11]. Every day postoperatively, the animals were treated once with carprofen 4 mg/kg subcutaneously (Rimadyl, Zoetis GmbH, Berlin, Germany) for analgesia. At the second follow-up, 7 days after surgery, ISQ evaluation and radiographic imaging were repeated.

The EDX and TEM analyses included six drill samples, one for each of the following temperatures: 4 °C, 3 °C, 2 °C, 48 °C, 49 °C, and 50 °C. The EDX analysis was performed using a scanning electron microscope (SEM; ESEM XL 30 FEG; FEI, Eindhoven, the Netherlands) equipped with an EDX detector system (EDAX, Mahwah, NJ) in the backscatter mode, with an acceleration voltage of 15 kV. The samples for the SEM and EDX analyses were fixed in 3% glutaraldehyde in 0.1 M Sorensen’s phosphate buffer, dehydrated in an ascending ethanol series (30%–100%), and dried at 37 °C, in accordance with the procedure used in a previously published study [6]. The EDX analysis was performed with an EDAX Genesis system (EDAX), at eight measurement points using a mean value for each sample.

For TEM, the samples were fixed in 3% glutaraldehyde in 0.1 M Sorensen’s phosphate buffer and decalcified in 0.5 M EDTA. After post-fixation in 1% OsO_4_ (Roth, Karlsruhe, Germany) in a 25 mM sucrose buffer, the samples were dehydrated in an ascending ethanol series, incubated in propylene oxide (Serva, Heidelberg, Germany) and embedded in Epon resin (Serva). Ultrathin Sects. (70–100 nm) were cut and stained with 0.5% uranyl acetate and 1% lead citrate (both EMS, Munich, Germany) for contrast enhancement. The samples were viewed at an acceleration voltage of 60 kV by using a Zeiss Leo 906 TEM service (Carl Zeiss, Oberkochen, Germany).

### Statistical analyses

Analyses were performed using the Prism 8 software for Mac OS X (GraphPad, La Jolla, CA) running on Apple OS X. The variables were analyzed using the Kolmogorov–Smirnov normality test. The Kruskal–Wallis and Dunn’s multiple comparison tests with adjustment were used to identify differences between the parameters.

Post hoc power analysis was performed with the G*Power software (Heinrich-Heine-Universität, Düsseldorf, Germany) using the post hoc analysis of variance with groups to determine a power of 100% (parameter primary study aim EDX) based on the total sample size of 30 and six groups using an effect size of 11.23 and an *α* of 0.05.

## Results

**Fig. 3:**
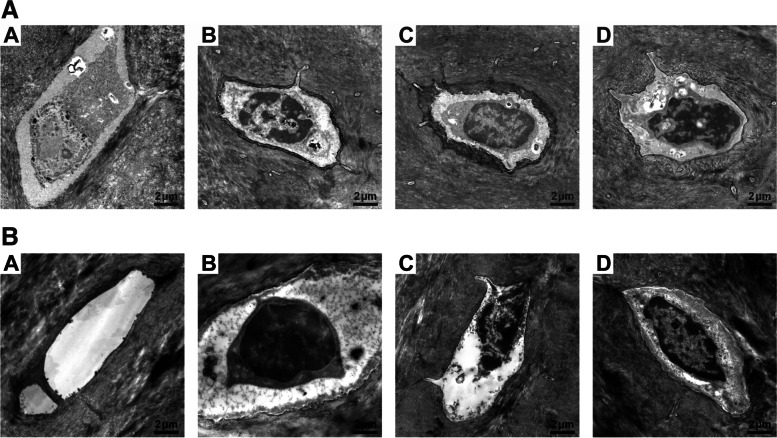
**A** TEM images of bone cells at cold temperatures. The osteocytes (O) in the lacunae (L) of the bone at 4 °C (A), 3 °C (B), and (C) 2 °C show acute damages. Some are necrotic with empty lacunae, whereas others vacuolized (arrowhead), shrank, and lost attachment (arrow) to the bone matrix (M). Each picture shows the most representative osteocyte of the respective sample. **B** TEM images of bone cells at warm temperatures. The osteocytes (O) in the lacunae (L) of the bone at 50 °C (A), (B) 49 °C, and (C) 48 °C show acute damages with necrosis, shrinkage, detachment, and vacuolization, similarly to those treated with cold temperatures. The degree of damage was worst at 50 °C, where almost all osteocytes were gone. Each image shows the most representative osteocyte of the respective sample

For the EDX analysis, one sample for each of the six temperature/time intervals was used in the evaluation (Table 1). A significant increase in bone calcium content was observed in the warm temperature groups (48 °C–50 °C, *p* < 0.01), which increased when the cold temperatures were reduced but without any significant difference within the group. By contrast, the carbon content decreased parallel to the increase in calcium content. The calcium-to-carbon ratio was used to normalize the calcium content to the organic component and therefore reflected the degree of bone calcification, which increased with increasing temperatures. Moreover, the sodium content increased significantly with the temperature stimulus but without significant differences between the cold and warm groups. Like the calcium content, the phosphate value analogously reflected increases in the values with increasing damaging temperature stimulus (3 °C–50 °C, *p* = 0.01 and 48 °C–50 °C, *p*< 0.01). The calcium-to-phosphate ratio increased with decreasing cold and increasing warm temperatures. The sulfur content decreased when the temperature was decreased from 4 °C to 2 °C and from 48 °C to 50 °C [12]. TEM revealed that at all the applied cold and warm temperatures, the osteocytes in the lacunae of the bone within the treatment area showed signs of cell damage, including vacuolization, shrinkage, and detachment from the surrounding bone matrix. Some cells became necrotic, leaving empty lacunae behind (Fig. 3). The TEM images of osteocytes showed that both heat and cold treatments induced cellular damage. The damage at hot temperatures (48 °C, 49 °C, and 50 °C) was greater than that at cold temperatures (2 °C, 3 °C, and 4 °C). At warm temperatures, the cell injury worsened with the increase in temperature applied such that 50 °C led to irreversible cell death. The degree of damage was greater at 50 °C and 2 °C than at 48 °C and 5 °C.

**Table 1:** Descriptive and statistical values for EDX analysis of bone composition between the groups

		4 °C		3 °C		2 °C		48 °C		49 °C		50 °C		*p* Value
**Weight %**	**Element**	**Mean**	**SD**	**Mean**	**SD**	**Mean**	**SD**	**Mean**	**SD**	**Mean**	**SD**	**Mean**	**SD**	
	Calcium	2.27	2.33	4.63	6.20	29.33	35.71	0.55	0.22	13.63	10.08	25.31	4.78	2°–48° *p* < 0.01 3°–50° *p* = 0.01
														4°–50° *p* < 0.01 48°–50° *p* < 0.01 48°–49° *p* < 0.01
	Carbon	86.34	4.12	85.83	7.29	57.56	34.48	89.90	2.44	74.05	13.06	48.70	6.89	4°–49° *p* = 0.04 4°–50° *p* < 0.01 3°–49° *p* = 0.04 3°–50° *p* < 0.01
	Oxygen	7.45	3.01	5.96	2.93	5.22	4.07	8.01	2.54	4.25	1.86	12.63	5.46	2°–50° *p* = 0.04 49°–50° *p* < 0.01
	Sodium	0.32	0.15	0.30	0.11	0.64	0.55	0.33	0.07	0.48	0.19	0.69	0.25	3°–50° *p* = 0.01
	Phosphate	2.09	2.82	2.33	2.76	6.51	5.79	0.43	0.34	7.09	4.18	12.40	1.98	2°–48° *p* = 0.01 3°–50° *p* = 0.01 4°–50° *p* < 0.01 48°–50° *p* < 0.01 48°–49° *p* < 0.01
	Sulphur	1.52	1.77	0.94	0.46	0.74	0.85	0.79	0.26	0.50	0.37	0.27	0.17	3°–50° *p* < 0.01 4°–50° *p* = 0.02 2°–48° *p* < 0.01
**Ratios**	Ca/C	0.03		0.05		0.32		0.01		0.18		0.5		
	Ca/P	1.09		1.98		3.14		1.27		1.92		2.04

**Fig. 4:**
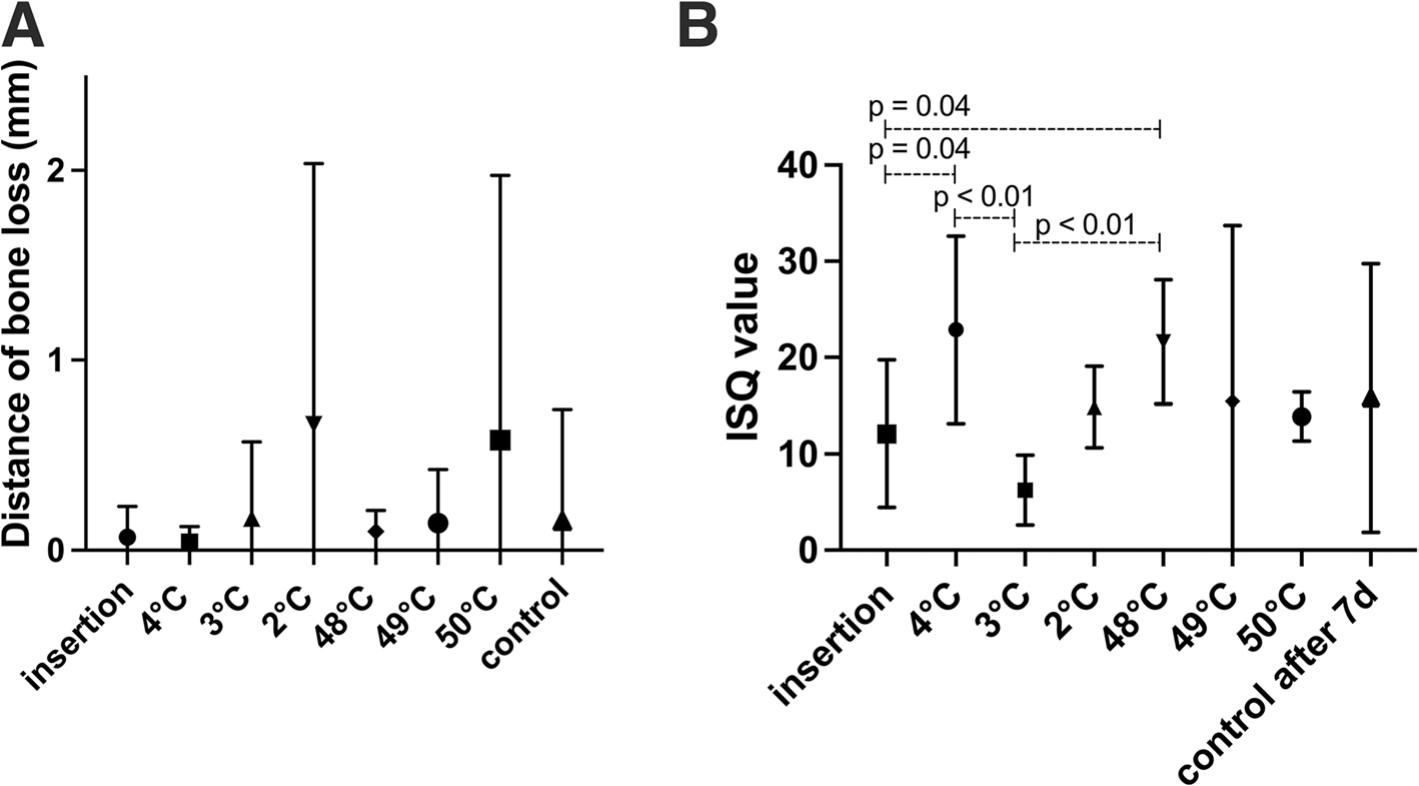
**A** The distance of the peri-implant bone loss is shown for the test and control groups after the 7-day follow-up. Furthermore, the measurement after implant insertion is presented. **B** The implant stability (ISQ) values are presented for the test and control groups after implant insertion and the 7-day follow-up

The measurement of peri-implant bone loss showed an increase in the distance from the bone attachment at the implant to the shoulder with increasing temperature stimulus. However, these differences were insignificant (*p* > 0.05; Fig. 4A).

The mean ISQ values reflect the same stability values for the implant placement and the control group after 7 days. An inhomogeneous result was evaluated in the test groups, as the ISQ values at 4 °C–3 °C decreased and those at 3 °C–2 °C increased again (*p* < 0.05). On the other hand, decreasing stability values were observed at temperatures of 48 °C–50 °C, but the differences were not statistically significant (Fig. 4B).

## Discussion

The impact of targeted heating or cooling was evaluated in this rat study to obtain evidence of the behavior of peri-implant bone necrosis for inducing implant removal. Eriksson and Albrektsson investigated the effects of heat on bone metabolism and published their data. They reported that arterial and venous hyperemia could be observed at short high-temperature surges, with a partial halt (hemostasis) of blood flow in numerous capillaries [13]. However, after 3 weeks, bone resorption was observed [13].

Mineral bone composition can indicate bone damage. Decreased Ca/P ratio is associated with induced bone loss [14], whereas the Ca/C ratio indicates the degree of calcification of the bone matrix at different stages in the respective measure [15]. These study results showed that both the Ca/P and Ca/C ratios increased with the worsening of the temperature-induced bone damage. In the damaged bone matrix, the Ca/C ratio also increased [15]. The warm temperature groups showed a substantial increase in bone calcium content, which also increased when the cold temperatures were reduced, but no significant within-group difference was found. In our study, the carbon content reflected the organic compound of the bone and decreased parallel to the increase in calcium content. Sulfur content, which is also correlated to bone physiology, decreased when the temperature decreased from 4 °C to 2 °C and from 48 °C to 50 °C [12].

The TEM images in another study showed that a temperature input of 53 °C resulted in decalcification and enlarged mitochondria, which lost their inner cristae structure [8]. In our investigation, warm temperatures also led to cell injury and irreversible cell death at 50 °C. Furthermore, the first-choice imaging modality should always be conventional radiography, as it provides an overview of the architecture and pathological states of the bone and soft tissues of the region of interest [16]. The distance between the implant shoulder and the bone contact with the implant was measured in this study to gather data on the behavior of the peri-implant bone level around zirconia implants [10]. Higher temperatures [17] led to not only loss of bone contact but also increases in the sizes of the infrabony pockets next to the treated implant. An increased distance over time may indicate bone loss and implant loosening. However, this study showed no significant differences over the 7-day follow-up period. In another study, the peri-implant pockets were significantly larger than those in the control group at 50 °C for 1 min [17]. A more extended follow-up for this parameter seems advisable for future study protocols. Furthermore, CT or magnetic resonance imaging might be more sensitive, as the sensitivity of plain film for detecting early stages of bone necrosis is rather low at 41% [18].

Oscillations occurring as reactions to the implant-to-bone contact can indicate the bone-to-implant contact interface from the resonance frequency analysis (RFA). The ISQ is the unit of measurement for RFA [19]. In humans with real implant geometries, the values range from 1 to 100, with a high ISQ score (> 60) indicating good stability [9]. In this study, the ISQ system was tested on miniature implants, as a suitable thread could be cut on the mini-screws. The aim was to determine whether such implant loosening can be detected in small animal models as well. As a critical reflection, we can infer that the ISQ model was established for real implants in humans. Therefore, we can assume that the stability values are fundamentally lower than those in humans owing to the smaller size. This was also shown in our evaluation and should be considered when interpreting our data. An inhomogeneous result was evaluated in the test groups; nevertheless, at temperatures ranging from 48 °C to 50 °C, decreasing stability values were found, but the differences were not significant. In the following pig study with real implants, based on these findings, the ISQ value measurement could be used to detect implant loosening.

Furthermore, the implants used in this study were not osseointegrated bodies. As a result, identifying valid threshold temperature values for osseointegrated implants necessitates in vivo research. With regard to implant survival there are no adverse results in diabetes type I patients [20]. However, in terms of possible complications, more postoperative bleeding and wound infections have been described. Regarding thermo-explantation, increased bleeding may affect the temperature level. As a successful thermo-explantation can only be confirmed in osseointegrated implants, this rat study was conducted to minimize the sample sizes in later animal studies using pigs with osseointegrated implants. As soon as the temperature time interval can be narrowed down, further studies will analyze the effect of different implant designs and shoulder formations on the described method [21]. If a special device can be developed, then for the current occasion a sufficient reduction of air contamination should also be considered [22].

## Conclusion

The cell damage at hot temperatures (48 °C, 49 °C, and 50 °C) was greater than that at cold temperatures (2 °C, 3 °C, and 4 °C). Although this was a preliminary study, the temperatures and intervals identified in areas of both heat and cold could help lower the number of samples in further studies of thermo-explantation. At a temperature of 50 °C at a time interval of 60 s, significant bone composition changes were observed. This level may be used for future thermo-explantation. The subsequent planned in vivo pig study, which will consider osseointegrated implants, is feasible.

## Supplementary information

**Additional file 1.**

**Additional file 2.**

## Data Availability

The datasets used and/or analyzed during the current study available from the corresponding author on reasonable request.
