# Supplementary material for: Results of thermal osteonecrosis for implant removal on electron microscopy, implant stability, and radiographic parameters – a rat study
Source: Head Face Med. 2023 Mar 7;19:4. doi: 10.1186/s13005-023-00349-2 (PMC9990269; doi:10.1186/s13005-023-00349-2)
Supplement: Supplementary file 2 — Additional file 2. [file 13005_2023_349_MOESM2_ESM.pdf]

Landesamt für Natur,
Umwelt und Verbraucherschutz
Nordrhein-Westfalen

LANUV NRW, Postfach 10 10 52, 45610 Recklinghausen

Herrn
Dr. Kristian Kniha
Klinik u Poliklinik für Mund-, Kiefer- und Gesichtschirurgie
Universitätsklinik RWTH Aachen
Pauwelsstraße 30
52074 Aachen

Auskunft erteilt:
Frau Wegener
Direktwahl 02361 305-3212
Fax 02361 305-3062
fachbereich81@lanuv.nrw.de

DE-MAIL:
fachbereich81@lanuv-nrw.de-mail.de

Aktenzeichen
81-02.04.2019.A276
bei Antwort bitte angeben

Ihre Nachricht vom:
Ihr Aktenzeichen:

Datum: 11.11.2019

Hauptsitz:
Leibnizstraße 10
45659 Recklinghausen
Telefon 02361 305-0
Fax 02361 305-3215
poststelle@lanuv.nrw.de
www.lanuv.nrw.de

Dienstgebäude:
Hauptsitz Recklinghausen

Öffentliche Verkehrsmittel:
Ab Recklinghausen Hbf mit Buslinie 236 oder 237 bis Haltestelle "LANUV" und 5 Min. Fußweg oder mit Buslinie SB 20 bis Haltestelle "Hohenhorster Weg" und 15 Min. Fußweg in Richtung Trabrennbahn bis Leibnizstraße

Tierversuche
Ihr Antrag auf Genehmigung eines Versuchsvorhabens gemäß § 8 Abs. 1 Tierschutzgesetz (TierSchG) i. V. m. § 31 Tierschutz-Versuchstierverordnung (TierSchVersV) vom 03.07.2019, eingegangen am 10.07.2019;
Rückfragen vom 27.09.2019
Ihre e-mail vom 23.10.2019

Sehr geehrter Herr Dr. Kniha,

I. Gemäß § 8 Abs. 1 Tierschutzgesetz (TierSchG) in der Fassung der Bekanntmachung vom 18.05.2006 (BGBL.I, S.1206,1313) in der derzeit geltenden Fassung i. V. m. § 33 der Verordnung zum Schutz von zu Versuchszwecken oder zu anderen wissenschaftlichen Zwecken verwendeten Tieren (Tierschutz-Versuchstierverordnung – TierSchVersV) vom 01.08.2013 (BGBl. I, 3125) in der derzeit gültigen Fassung erteile ich Ihnen, unter dem Vorbehalt des jederzeitigen Widerrufs, die Genehmigung zur Durchführung des Tierversuches mit dem Titel:
"Osseodisintegration enossaler Implantate mit biophysikalischen Methoden (ODIN)".

Bankverbindung:
Landeshauptkasse NRW
Helaba
BIC-Code: WELADEDD
IBAN-Code:
DE 41 3005 0000 0004 1000 12

II. Die Verantwortung für die Durchführung des Tierversuchsvorhabens obliegt folgenden Personen:
Verantwortliche(r) Leiter(in): Herr Dr. Evgeny Goloborodko
Stellvertretende(r) Leiter(in): Herr Dr. Ali Modabber

III. An der Durchführung des Tierversuchsvorhabens dürfen neben der/dem Leiter(in) und der/dem Stellvertreter(in) folgende Personen beteiligt werden:

1. Herr Dr. Kristian Kniha
2. Herr Dr. Stephan Möhlhenrich
3. Herr Dr. Marius Heitzer
4. Frau Britta Bungardt

IV. Die Versuche dürfen nur in den Räumlichkeiten im Institut für Versuchstierkunde der Medizinischen Fakultät der RWTH Aachen, 52074 Aachen durchgeführt werden.

V. ☒ Eine rückblickende Bewertung nach § 35 TierSchVersV ist nicht vorzunehmen.

☐ Das Versuchsvorhaben ist gemäß § 35 Abs. 1
Satz 1 TierSchVersV ☐
Satz 2 Nr. 1 TierSchVersV ☐
Satz 2 Nr. 2 TierSchVersV ☐
Satz 2 Nr. 3 TierSchVersV ☐
rückblickend zu bewerten.

VI. Die Genehmigung erstreckt sich auf Versuche mit:

Tierart		Anzahl
**Ratte**	:	**64**
**Schwein**	:	**10**

VII. Sie haben folgende Auflagen zu beachten:

1. Bitte teilen Sie vor Versuchsbeginn die aus den Vorstudien ermittelten Temperierungsintervalle und Temperierungsquellen mit.

2. Bitte nennen Sie vor Beginn des Versuchs 2 die Zeitfenster bzw. teilen Sie mit, ob diese sich aufgrund von Versuch 1 verschieben.
3. Ist ein Transport der Versuchstiere zwischen Operations- und Tierhaltungsraum unvermeidbar, so ist dafür Sorge zu tragen, dass mit Hilfe geeigneter Behältnisse dieser Transport so durchgeführt wird, dass negative Beeinflussungen durch äußere Einflüsse (z.B. Witterung, Lärm, etc.) ausgeschlossen sind. Es ist insbesondere darauf zu achten, dass die Tiere keinen Temperaturschwankungen ausgesetzt sind.
4. Unabhängig von den im Antrag angeführten Abbruchkriterien ist der Versuch abzubrechen und das betroffene Tier tierschutzgerecht zu töten, wenn dies aufgrund des Zustandes des Tieres nach der Einschätzung der/des Tierschutzbeauftragten aus Tierschutzgründen unerlässlich ist.
5. Bitte teilen Sie nach Erhalt der Genehmigung mit, ob die von Ihnen eingestellte Nichttechnische Projektzusammenfassung (NTP) immer noch den Inhalt der Genehmigung vollständig und richtig darstellt und somit zur Veröffentlichung freigegeben werden kann. Andernfalls aktualisieren und übersenden Sie bitte die NTP mit der neuen ID-Nummer.

VIII. Diese Genehmigung ist bis zum 30.11.2024 befristet.

IX. Für diesen Bescheid wird eine Gebühr erhoben. Hierzu ergeht ein gesonderter Gebührenbescheid.

Erläuterungen und Begründung:

Zu I.:
Der Inhalt Ihrer Antragsunterlagen ist Gegenstand dieser Genehmigung. Jede Änderung ist dem Landesamt für Natur, Umwelt und Verbraucherschutz NRW (LANUV) unverzüglich - unter Angabe des Aktenzeichens - mitzuteilen (§ 34 Abs. 1 Nr. 4 TierSchVersV).

Es wird empfohlen, allen an der Tierversuchsdurchführung beteiligten Personen diese Genehmigung zur Kenntnis zu geben.
Die Tierschutzbeauftragte, Frau Dr. Kira Scherer, und das zuständige Veterinäramt der Städteregion Aachen erhalten eine Durchschrift dieser Genehmigung.

Der Widerruf dieser Genehmigung kann erfolgen, wenn gegen diese Genehmigung verstoßen wird.
Außerdem kann die Einstellung des Tierversuchs gemäß § 16a Abs. 1 Nr. 4 TierSchG angeordnet werden, wenn Tierversuche ohne die erforderliche Genehmigung oder entgegen tierschutzrechtlicher Bestimmungen durchgeführt werden.

Zu II.:
Für die Einhaltung der tierschutzrechtlichen Vorschriften zur Durchführung von Tierversuchen ist der bzw. die oben bezeichnete Leiter/in des Tierversuchsvorhabens oder im Falle deren/dessen Verhinderung ihr/sein in dieser Genehmigung bezeichnete/r Vertreter/in verantwortlich (§ 30 TierSchVersV).
Jeder beabsichtigte Wechsel der/des Versuchsleiterin/-leiters oder der Stellvertreterposition ist dem Landesamt für Natur, Umwelt und Verbraucherschutz NRW unverzüglich anzuzeigen (§ 34 Abs. 2 TierSchVersV).

Zu III.:
Die bezeichneten Personen dürfen ausschließlich die im Genehmigungsantrag aufgeführten Eingriffe oder Behandlungen im Rahmen der zulässigen Verantwortlichkeitsstufe durchführen. Zu beachten ist, dass Personen, die Eingriffe und Behandlungen innerhalb des Versuchsvorhabens durchführen sollen und die Voraussetzungen nach § 16 Abs. 1 Sätze 2 und 3 TierSchVersV nicht erfüllen, erst nach Erteilung einer Ausnahmegenehmigung gem. § 16 Abs. 1 Satz 5 TierSchVersV durch die zuständige Kreisordnungsbehörde eingesetzt werden dürfen.

Zu VIII.:
Die Genehmigung ist gem. § 33 Abs. 2 Satz 1 TierSchVersV auf höchstens fünf Jahre zu befristen.
Sofern die Befristung antragsgemäß weniger als fünf Jahre beträgt, kann ein Antrag auf Verlängerung gestellt werden. Der Antrag ist rechtzeitig vor Ablauf der Genehmigungsfrist – über den/die zuständige/n Tierschutzbeauftragte/n – zu stellen. Die Verlängerung kann gem. § 33 Abs. 2 Satz 2 TierSchVersV höchstens zweimal um jeweils bis zu einem Jahr erfolgen, wobei die Gesamtdauer der Genehmigung von fünf Jahren nicht überschritten werden darf.

Landesamt für Natur,
Umwelt und Verbraucherschutz
Nordrhein-Westfalen

Hinweis:
Eventuell erforderliche weitere Genehmigungen nach dem Tierschutzgesetz oder anderen gesetzlichen Bestimmungen bleiben von dieser Genehmigung unberührt.

Rechtsbehelfsbelehrung:
Gegen diesen Bescheid kann innerhalb eines Monats nach Bekanntgabe Klage beim Verwaltungsgericht Aachen, Kasernenstraße 25, 52064 Aachen (Postfach 101051, 52010 Aachen), erhoben werden.

Die Klage ist schriftlich beim Verwaltungsgericht einzureichen oder zur Niederschrift der Urkundsbeamten der Geschäftsstelle zu erklären.

Die Klage kann auch durch Übertragung eines elektronischen Dokuments an die elektronische Poststelle des Gerichts erhoben werden. Das elektronische Dokument muss für die Bearbeitung durch das Gericht geeignet sein. Es muss mit einer qualifizierten elektronischen Signatur der verantwortenden Person versehen sein oder von der verantwortenden Person signiert und auf einem sicheren Übermittlungsweg gemäß § 55a Absatz 4 VwGO eingereicht werden. Die für die Übermittlung und Bearbeitung geeigneten technischen Rahmenbedingungen bestimmen sich nach näherer Maßgabe der Verordnung über die technischen Rahmenbedingungen des elektronischen Rechtsverkehrs und über das besondere elektronische Behördenpostfach (Elektronischer-Rechtsverkehr-Verordnung - ERVV) vom 24. November 2017 (BGBl. I S. 3803).

Mit freundlichen Grüßen
Im Auftrag

A. Sommerfeld

Dr. Annika Sommerfeld
